# Cross-Compartment Virome Profiling in Human Immunodeficiency Virus Infection and Substance Use Disorder Reveals Brain–CSF–Periphery Discordance and Hepatitis B Virus in Central Nervous System

**DOI:** 10.3390/ijms27125349

**Published:** 2026-06-13

**Authors:** Xin Dang, Barbara A. Hanson, Melissa Lopez, Janet Miller, Igor J. Koralnik

**Affiliations:** 1 Davee Department of Neurology, Feinberg School of Medicine, Northwestern University, Chicago, IL 60611, USA; xin.dang@northwestern.edu (X.D.); barbara.hanson@northwestern.edu (B.A.H.); melissa.lopez@northwestern.edu (M.L.); 2 Northwestern Medicine, Chicago, IL 60611, USA; jmiller@nm.org

**Keywords:** ViroFind, metagenomic sequencing, viruses, cerebrospinal fluid, brain

## Abstract

The diversity and abundance of the brain virome is an active field of investigation. However, how the brain virome relates to the presence of viruses outside of the nervous system remains unclear. The rationale for this study is that analyses across multiple biologically linked compartments within the same individuals provide an important opportunity to evaluate virome discordance and viral burden. To characterize viral prevalence and burden across anatomical compartments, we applied the targeted viral enrichment method ViroFind to matched postmortem brain (*n* = 66), cerebrospinal fluid (CSF; *n* = 24), and peripheral samples (spleen, peripheral blood mononuclear cells, and lymph nodes; *n* = 66) from individuals with and without human immunodeficiency virus (HIV) infection and substance use disorder (SUD) in the National NeuroAIDS Tissue Consortium. We detected nucleic acids from 27 viruses representing 12 taxa. Several viruses, including adenovirus, torque teno virus, Epstein–Barr virus, human herpesvirus 6 and 7, cytomegalovirus, parvovirus, and JC polyomavirus, showed significant inter-compartment differences in prevalence or burden. CSF exhibited lower overall viral diversity than brain or peripheral samples, whereas peripheral samples showed the highest viral burden. CNS viral detection was more likely when the same virus was also detected in the periphery. We also detected HBV and HCV in CNS samples despite them not being classically regarded as neurotropic. Broader virome profiling showed greater peripheral viral burden and diversity in HIV-positive than HIV-negative individuals, whereas SUD was not associated with overall viral burden differences. These findings highlight important cross-compartment differences in viral detection, including occurrence of occult HBV infection within the CNS, and support the value of CNS–periphery comparisons in virome studies. These findings can contribute to improved diagnosis and management of viral infections.

## 1. Introduction

Metagenomic next-generation sequencing (mNGS) enables comprehensive profiling of viral and bacterial populations within clinical samples and is increasingly utilized in diagnostic and research settings. While prior studies have leveraged mNGS to examine accessible fluid compartments, including cerebrospinal fluid (CSF) [1,2,3,4], blood [5], plasma [6,7,8], serum [9], cervical swabs [10], bronchoalveolar lavage [11,12], and semen [13], these approaches often fail to capture viruses present at low abundance or in less accessible tissues. Some prior protocols have relied on rolling-circle amplification, which enriches circular DNA viruses but is less likely to detect other viral taxa [7,10]. Moreover, few have applied viral enrichment techniques during library preparation, limiting sensitivity for low-level viral populations and reducing the ability to compare matched brain, CSF, and peripheral compartments within the same individuals. In this study, the peripheral comparator includes PBMC, spleen, and lymph node specimens, which differ in cellular composition and viral ecology and should therefore be interpreted as a heterogeneous pooled compartment.

We previously characterized the brain virome in postmortem samples from the National Neuro-HIV Tissue Consortium (NNTC), comprising individuals infected with HIV and/or with substance use disorder (SUD), using a targeted enrichment viral capture method, ViroFind [14]. That report revealed the presence of nucleic acids from bloodborne viruses, including Hepatitis B virus (HBV) and Hepatitis C virus (HCV), in central nervous system (CNS) tissue. Increasing evidence suggests that bloodborne viruses may persist in the CNS without overt symptoms of neuroinfection, particularly in the setting of chronic infections and altered host immunity. In addition, we have recently investigated the predictive utility of ViroFind detection of peripheral and CSF viromes for viral presence in human brain tissue [15].

The present study extends that work by examining matched peripheral and CSF samples from our previously studied participants to better understand the anatomical distribution, cross-compartment detection patterns, and associations of viral detection with HIV and SUD status across brain, CSF, and peripheral compartments. In doing so, we sought to identify viruses associated with HIV or SUD status, patterns of concordant and discordant detection, and to examine cases in which viral nucleic acids were detected in CNS compartments despite negative peripheral testing. Analyses incorporating multiple biologically linked compartments within the same individuals provide a rare opportunity to evaluate virome discordance and viral burden stratified by HIV and SUD status. This paired-compartment approach allows for a more granular assessment in the setting of HIV and SUD. In this study, we characterize cross-compartment virome profiles in brain, CSF, and peripheral samples and identify key features of viral concordance, discordance, and findings of HBV in the CNS and periphery of people with and without HIV and SUD.

## 2. Results

### 2.1. Samples

A total of 72 brain samples from the NNTC were analyzed with ViroFind. The results of this study, demographics, and type of drug usage were previously stated in [14]. Of those, 66 matched peripheral samples (63 spleen, 2 lymph node, 1 PBMC; collectively referred to as “periphery” throughout) and 24 matched CSF samples were obtained. Because the periphery pools PBMC, spleen, and lymph node specimens, comparisons with brain and CSF should be interpreted cautiously. Among the CSF subset, research group 1 (HIV^+^/SUD^+^) had 8 samples and group 2 (HIV^+^/SUD^−^) had 12 samples, while group 3 (HIV^−^/SUD^+^) only had three samples, and group 4 (HIV^−^/SUD^−^) only had one sample. Because of this limitation, analyses comparing HIV^+^ vs. HIV^−^ status were not performed for the CSF compartment. SUD group status attribution was performed by the NNTC staff. Qualitative drug use data were collated by the NNTC and consisted of multiple urine screening and neuropsychiatric evaluations (CIDI/PRISM) [16,17]. A summary of urine screening and PRISM-defined psychiatric substance use diagnoses for the SUD^+^ groups is provided in Table 1. Urine screening identified cannabis, opiates, cocaine, benzodiazepines, amphetamine/methamphetamine, PCP, barbiturates, methadone, and other substances, while PRISM diagnoses captured substance use categories, including cannabis, opiates, stimulants, sedatives, hallucinogens, alcohol, and other substances. Overall, urine screening showed a median of one substance detected per subject, with a higher number of substances detected in the HIV^+^/SUD^+^ group than in the HIV^−^/SUD^+^ group. Cocaine and cannabis detection were also more frequent in the HIV^+^/SUD^+^ group, while methadone was detected only in the HIV^−^/SUD^+^ group. PRISM-based diagnoses showed a median of three substance use diagnoses overall, with cocaine, alcohol, cannabis, and opiate diagnoses being the most common categories. Due to polysubstance use, no attempts to stratify drug usage by class were made.

### 2.2. Characterization of Viral Species Detected in Matching Brain, CSF, and Peripheral Samples and Matching Brain and Peripheral Compartments

**Figure 1:**
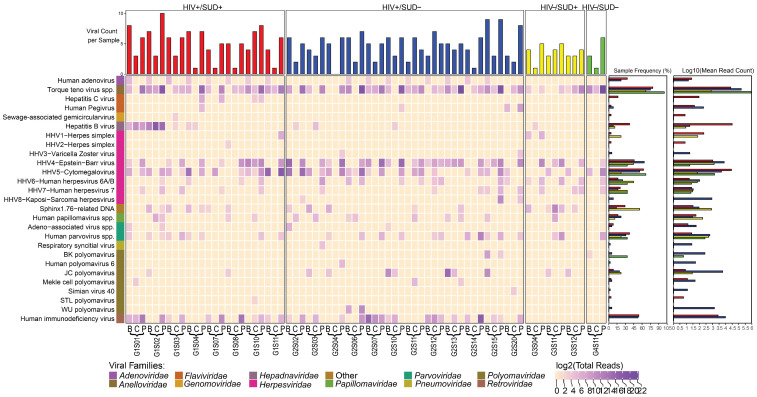
Heatmap of matching brain, CSF, and peripheral virome by individual.

**Figure 2:**
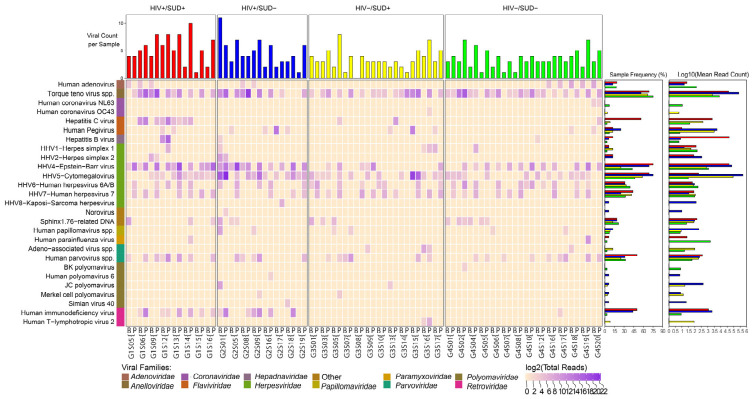
Heatmap of matching brain and peripheral virome by individual.

We first evaluated the overall virome landscape across brain, CSF, and periphery to establish baseline patterns of viral presence and burden across compartments. This global comparison provides the structural context for subsequent taxon-specific and compartment-specific analyses. Viral species were analyzed in donors with matching samples from three (brain, CSF, and periphery; Figure 1) or two (brain and periphery; Figure 2) compartments. The study design incorporated two different categorical variables (HIV and SUD) which collectively are sub-divided into four research groups (HIV^+^/SUD^+^, HIV^+^/SUD^−^, HIV^−^/SUD^+^, HIV^−^/SUD^−^). Primary ViroFind results are presented in heatmaps summarizing viral detection across compartments for brain, CSF, and periphery triple comparison (Figure 1; B-brain, C-CSF, and P-periphery), and brain, periphery double comparison (Figure 2; B-brain, and P-periphery). We detected nucleic acids from 27 viruses belonging to 12 viral taxa.

“B” represents brain, “C” is for CSF, and “P” is for peripheral samples (spleen, PBMC, and lymph node). All samples are divided into four categories: HIV^+^/SUD^+^ (red, *n* = 8), HIV^+^/SUD^−^ (blue, *n* = 12), HIV^−^/SUD^+^ (yellow, *n* = 3), and HIV^−^/SUD^+^ (green, *n* = 1), shown in the computed heatmap above showing all viral taxa identified by the ViroFind in-house pipeline with purple log2 gradient scale indicating the raw number of viral reads. The frequency of each viral species, as well as the raw mean read count on a log scale for both groups are also shown. Colors in the top and side bar graphs indicate study group: HIV^+^/SUD^+^ (red), HIV^+^/SUD^−^ (blue), HIV^−^/SUD^+^ (yellow), and HIV^−^/SUD^−^ (green).

“B” represents brain and “P” peripheral samples (spleen, PBMC, and lymph node). All samples are divided into four categories: HIV^+^/SUD^+^ (red, *n* = 8), HIV^+^/SUD^−^ (blue, *n* = 8), HIV^−^/SUD^+^ (yellow, *n* = 12), and HIV^−^/SUD^+^ (green, *n* = 14), shown in the computed heatmap showing all viral taxa identified by the ViroFind in-house pipeline with purple log2 gradient scale indicating the raw number of viral reads. The frequency of each viral species, as well as the raw mean read count on a log scale for both groups are also shown. Colors in the top and side bar graphs indicate study group: HIV^+^/SUD^+^ (red), HIV^+^/SUD^−^ (blue), HIV^−^/SUD^+^ (yellow), and HIV^−^/SUD^−^ (green).

### 2.3. Comparison of Viral Prevalence and Burden in Brain, CSF, and Peripheral

For the 24 donors with brain, CSF, and peripheral samples available, we compared viral prevalence and cumulative burden across the three sampled compartments (Table 2). Both the number of viral species per donor and total viral burden differed significantly among compartments (*p* < 0.001). In general, viral prevalence and burden were lowest in CSF and highest in the periphery. Among individual taxa, Adenovirus (Adv), TTV, EBV/HHV4, CMV/HHV5, HHV6A/B, HHV7, Sphinx 1.76-related DNA, parvovirus, and JC polyomavirus (JCV) differed significantly in prevalence, while TTV showed significant inter-compartment differences in viral burden, which was significantly higher in periphery than in CNS samples.

A reorganization of Figure 1, by matching brain, CSF, and periphery compartments, is shown in Figure S1.

### 2.4. Viral Detection Across CNS and Periphery Compartments

We next evaluated all viruses to quantify the probability of detection of a virus in the CNS (brain and/or CSF) if it was present or absent from the periphery. For all viruses with sufficient prevalence to permit statistical evaluation (*n* ≥ 2 in both CNS and periphery), we assessed (i) concordance: the probability of CNS detection given periphery positivity, (ii) discordance: the probability of CNS detection given periphery-negativity, and (iii) risk difference: the difference in probabilities of CNS detection between the periphery-positive vs. periphery-negative for each virus (Table 3). When adjusting for multiple comparisons, HCV (padj < 0.001), HIV (padj < 0.001), and HBV (padj = 0.013) were highly periphery concordant, with a much greater probability of viral detection in the CNS if the virus was also present in the periphery. No viruses had a higher probability of being detected in the CNS if it was absent in periphery as indicated by positive risk differences. Besides the HIV-infection status of study participants, none of the clinical measurements, including viral serologies, were known by the investigators prior to carrying out ViroFind analyses, which were performed blinded to these clinical data.

HIV neurotropism and neurovirulence have been extensively studied elsewhere. HBV and HCV showed strong concordance between CNS and periphery. All HBV periphery-positive individuals were HBV CNS-positive, while 13% of HBV periphery-negative cases were CNS-positive (*p* = 0.013). In addition, 45% of HCV periphery-positive individuals were HCV CNS-positive, compared to none of periphery-negative cases (*p* < 0.001). Although both viruses are primarily hepatotropic, their detection in CNS tissue indicates discordant cross-compartment viral detection in some donors.

### 2.5. HIV Suppression Status Affects Virome Burden and Diversity

**Figure 3:**
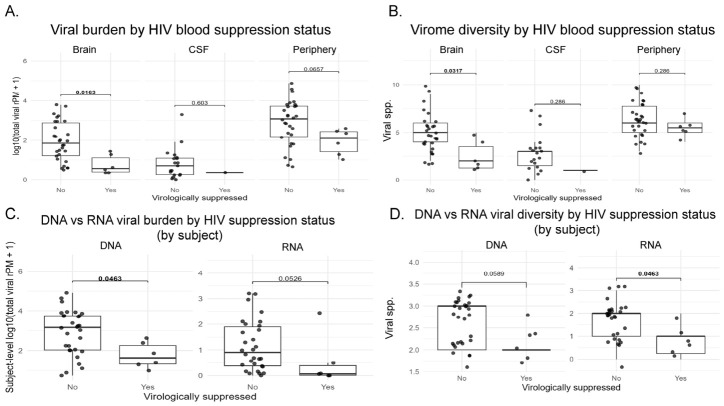
HIV suppression status in blood is associated with reduced virome burden and diversity in brains of HIV-positive individuals. (**A**) Total viral burden by compartment, calculated as log10(total viral rPM + 1). (**B**) Viral diversity by compartment, defined as the number of viral species detected per specimen. (**C**) Subject-level viral burden summarized by broad viral genome class, comparing DNA viruses and all RNA viruses. (**D**) Subject-level viral diversity summarized by RNA/DNA viral genome class. Boxplots show the median and interquartile range, with individual specimens (**A**,**B**) or subjects (**C**,**D**) shown as points. BH-adjusted *p*-values are shown above each comparison; bold values indicate padj < 0.05. rPM: reads per million. Spp.: species.

ART exposure history was not uniformly available; therefore, we performed an exploratory analysis using the available clinical suppression of HIV replication in blood among HIV^+^ individuals in Groups 1 and 2. Individuals with suppressed HIV replication showed lower viral diversity and total viral burden in brain specimens compared with non-HIV-suppressed individuals (Figure 3A,B). Overall subject-level viral diversity and total viral burden showed similar directional trends, although these comparisons did not remain significant after multiple-testing correction. When viral burden was summarized by genome class, HIV-suppressed individuals showed lower DNA viral burden and lower RNA viral diversity in the broad DNA/RNA analysis; however, this pattern was not retained when RNA viruses were separated into non-retroviral and retroviral groups (Figure 3C,D). Individual virus detection frequencies were not associated with HIV suppression status. These findings suggest that HIV suppression status may influence virome composition, particularly in brain specimens and broad viral genome-class summaries.

### 2.6. Comparison of Hepatitis B Virus Detection in the CNS and Periphery

**Figure 4:**
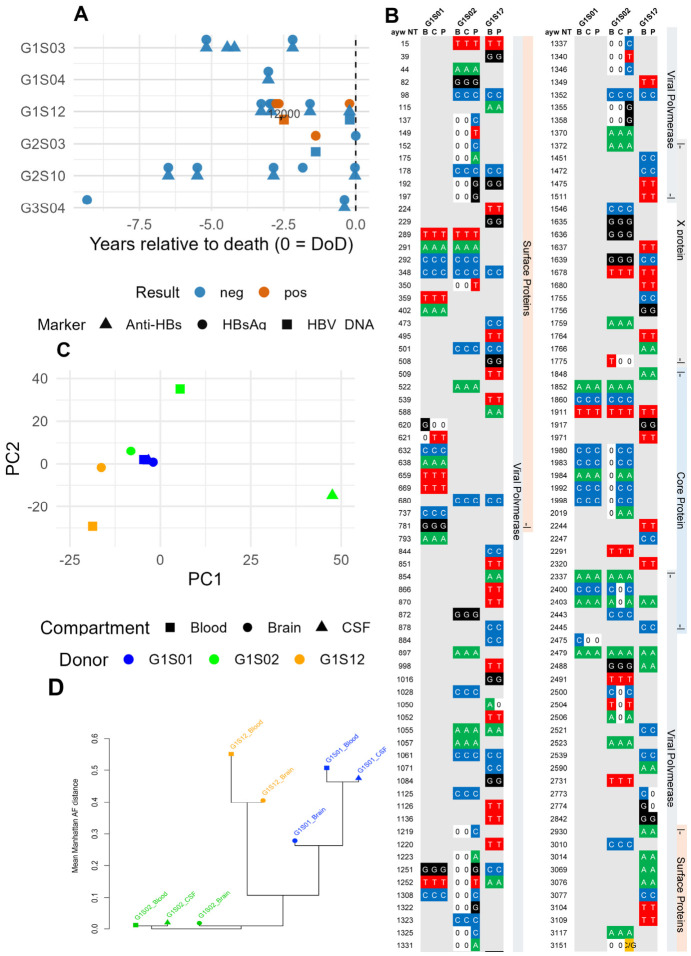
HBV serology and cross-compartment genomic variation across brain, CSF, and peripheral samples. (**A**) Serology timelines showing HBsAg (**top**), anti-HBs (**middle**), and HBV DNA (**bottom**) results by donor relative to death (0 = date of death [DoD]). Colors indicate positive (orange) and negative (blue) results. Viral load (cp/mL) for positive HBV DNA is indicated. (**B**) HBV genome map showing single-nucleotide polymorphisms (SNPs; >20% allele frequency, depth > 5) identified in brain (B), CSF (C), and periphery (P) compartments, relative to the HBV-ayw reference (NC_003977.2). SNPs were called only at loci with coverage ≥ 5 reads in all available compartments for that donor. Colored boxes indicate dominant alternative bases (adenine = green, cytosine = blue, thymine = red, guanine = black). Gray regions lack coverage or show no SNPs across compartments. Median coverage of 89.79% at depth > 5. (**C**,**D**) Principal component (PCA) and neighbor-joining phylogenetic tree analyses of allele frequencies (AF) (>1% alternative, depth > 10) across compartments. Samples cluster by donor, indicating lower intra-donor than inter-donor variation. Samples colored by donor, compartment of origin labeled by symbols (blood—square, brain—circle, CSF—triangle). Nucleotide colors indicate the dominant alternative base at each position: adenine (A, green), cytosine (C, blue), thymine (T, red), and guanine (G, black), while the colored side annotations indicate the corresponding HBV coding regions, including polymerase, surface, X, and core.

HBV was detected in CNS samples from this cohort more frequently than anticipated. We identified two subsets, CNS^+^/P^+^ and CNS^+^/P^−^, demonstrating discordant patterns of HBV detection across CNS and peripheral compartments (Figure 1 and Figure 2). Clinical HBV testing was longitudinally available for 6 of 11 (54.5%) donors with at least one HBV-positive compartment, allowing further comparison of ViroFind findings with available serologic HBV testing (Table 4; Figure 4). Because serologic data were available for only 6 of 11 donors with HBV detected in at least one compartment, classification of HBV detection patterns is limited in the remaining cases. Serologic testing revealed a range of profiles for HBsAg antigenemia, anti-HBs antibody, and circulating HBV DNA (Figure 4A). Occult HBV infection is conventionally defined by the presence of HBV DNA, typically in the liver, in the absence of detectable HBsAg in blood [18,19]. Applying this definition cautiously, we identified two donors (G2S03 and G2S10; Figure 4A, Table 4) in whom HBV DNA was detected in CSF, despite being negative for HBsAg testing in blood on the date of death, but HBV DNA-positive in CSF.

A third donor (G3S04) showed HBV DNA detection in postmortem CSF despite prior negative HBsAg testing, although the absence of testing on the date of death limits classification. A survey of Baltimore injection drug users [20] reported HBV DNA detection in CNS-related compartments even in the absence of antigenemia, which is consistent with our results. Sampling limitations preclude definitive statements about viral localization or replication capacity, and these findings are therefore best interpreted as discordant cross-compartment HBV detection.

### 2.7. HBV Sequence Diversity Across Compartments

HBV SNP detected at allele frequencies exceeding 20% in any compartment with coverage greater than five reads are shown in Figure 4B. This map highlights loci where at least one compartment (brain, CSF, or periphery) exhibited a robust alternative base call relative to the HBV-ayw reference genome (GenBank NC_003977.2). Regions corresponding to viral polymerase, surface, core, and X proteins are annotated to illustrate the distribution of polymorphisms across the HBV genome. The 20% threshold was selected to restrict visualization to high-confidence sites likely to represent true variants rather than sequencing noise. Allele frequency (AF) values from all callable sites (>1% alternative frequency, depth > 10 reads) were then used for principal component analysis and a neighbor-joining phylogenetic tree (PCA; Figure 4 C,D). Samples clustered more closely by donor than by compartment, showing that intra-donor variation was smaller than inter-donor variation, indicating that HBV sequence variation was more similar within donors than between donors.

Two donors (G1S02 and G1S03) had sufficient coverage in CSF to compare CSF-derived sequence to brain- and periphery-derived sequences. Because the CSF is a paucicellular compartment containing very few cells to support local viral replication, virorachia may reflect viral material originating from either the brain parenchyma or the peripheral blood. We therefore compared HBV allele frequency (AF) patterns across CSF, brain, and periphery in these donors. AF data were further restricted to loci showing both significant inter-compartment discordance (Fisher’s exact *p* < 0.05) and an absolute AF difference of at least 10%. Each allele site was classified as “brain-closer” or “periphery-closer” according to which compartment’s AF most closely matched that of the CSF. Under these criteria, donor G1S02 showed a significant predominance of brain-closer sites (34 brain-closer alleles vs. 13 periphery-closer alleles; *p* = 0.003), while donor G1S01 showed the opposite, non-significant trend (1 brain-closer allele vs. 4 periphery-closer alleles; *p* = 0.38). G1S12 did not have CSF available and was not included in the analysis.

These HBV findings highlight that HBV can be detected in CNS and peripheral compartments, including in some donors with negative peripheral testing.

### 2.8. Peripheral Viral Prevalence and Viral Burden Comparison of HIV^+^ vs. HIV^−^ Group

We next compared peripheral virome profiles between HIV^+^ and HIV^−^ donors to assess systemic viral burden. In the peripheral samples, we detected nucleic acid from 27 viruses belonging to 12 distinct taxa. HIV itself was excluded from statistical analyses. As shown in Table 5 and Figure S2, individuals who were HIV^+^ had a significantly higher frequency of viral prevalence and burden of viral species detected per than the HIV^−^ group (*p* < 0.001). Median species per subject was greater in HIV^+^ group (5 [IQR 4–7]) as compared to HIV^−^ subjects (4 [IQR 3–5]; *p* = 0.016, Mann–Whitney U test). Similarly, the median cumulative viral burden was higher in HIV^+^ samples (397.8 rPM [IQR 64.0–4179.2]) compared with HIV^−^ samples (17.0 rPM [IQR 6.6–64.6]; *p* < 0.001, Mann–Whitney U test). At the individual taxon level, significant differences were observed for EBV/HHV4 (*p* = 0.004, Fisher’s exact test for prevalence; *p* < 0.001, Mann–Whitney U for rPM) and CMV/HHV5 (*p* = 0.006 for prevalence; *p* = 0.050 for rPM). TTV also showed higher viral burden in HIV^+^ samples (*p* = 0.002). These findings indicate that HIV infection is associated with both broader viral diversity and higher circulating viral burden in periphery.

### 2.9. Periphery and CSF Viral Prevalence and Burden by SUD Status

We then evaluated whether SUD status was associated with differences in viral prevalence or burden in peripheral samples or CSF. As shown in Table 6 and Figure S3, overall viral burden did not differ significantly between SUD^+^ and SUD^−^ groups in the periphery. However, HCV (*p* = 0.002), and HHV7 (*p* = 0.041) showed higher prevalence among SUD^+^ donors, while EBV/HHV4 had a higher prevalence in SUD^−^ donors (*p* = 0.037; Table 5). HBV was not significantly more frequent in SUD^+^ peripheral samples (*p* = 0.098). In matched CSF samples (Table 7; Figure S4), no significant differences in viral prevalence or burden were observed between SUD^+^ (*n* = 11) and SUD^−^ (*n* = 13) donors.

## 3. Discussion

This study identifies cross-compartment differences in viral detection across brain, CSF, and periphery in a cohort of HIV^+^ and/or SUD-affected individuals. We extend prior work demonstrating HBV and HCV nucleic acids in CNS tissue by evaluating matched peripheral and CSF compartments and identifying instances of viral detection in the CNS despite negative peripheral testing. Among HBV-positive cases with available clinical testing, some donors showed HBsAg testing in blood, including one donor who met conventional blood-based criteria for occult HBV infection [19,20,21,22,23]. Among donors with available serologic data, two showed HBV DNA detection in CSF despite negative HBsAg testing and without concurrent HBV detection in paired brain or peripheral samples, while a third showed HBV DNA detection in both brain and the periphery despite HBsAg negativity at the time of final clinical testing. These cases illustrate discordance between blood-based HBV testing and HBV detection in CNS compartments. Allele frequency-based comparisons between CSF, brain, and the periphery revealed donor-specific differences, with one individual showing closer similarity between CSF and brain, and another showing no such pattern. These results suggest heterogeneity in HBV sequence relationships across compartments and individuals, precluding general conclusions at this stage.

Other viral taxa such as JCV and HIV have shown CNS-specific sequence evolution, including mutations associated with altered tropism or enhanced pathogenicity within the brain [24,25]. Whether HBV or HCV show comparable CNS-associated sequence patterns remain unknown. The present dataset provides preliminary evidence of viral nucleic acid presence in CNS compartments but is insufficient to assess replication competence, cell-type specificity, or CNS-adaptive sequence variation. Future studies with broader sampling and higher-resolution molecular characterization will be necessary to clarify the biological significance of HBV and HCV detection in CNS compartments.

Beyond HBV, we identified brain–periphery discordance in several viral taxa, most notably HPgV, AAV, and CMV/HHV5, suggesting that cross-compartment viral detection patterns may extend beyond HBV in chronic HIV/SUD contexts. Across the cohort, CSF samples exhibited lower overall viral burden and species diversity than peripheral or brain samples at the group level. In this cohort, CSF showed lower viral burden and species diversity than brain or periphery, and HIV infection was associated with significantly higher peripheral viral burden and diversity, while SUD status had more modest effects, limited primarily to prevalence differences for selected taxa in peripheral samples. In an exploratory analysis of HIV^+^ donors with available clinical virologic suppression data, suppressed individuals showed lower viral diversity and total viral burden in brain specimens, with similar directional trends at the subject level, suggesting that the suppression of HIV may influence virome composition, but requiring cautious interpretation given the limited number of suppressed donors.

This study is subject to several important limitations. First, the cohort size, particularly for matched CSF and brain samples, was modest, limiting statistical power and generalizability. CSF specimens were available for only one-third of the cohort and were unequally distributed across HIV and SUD subgroups, precluding certain direct comparisons. Similarly, SUD status was analyzed as a broad variable since the cohort was not powered to stratify by substance classes. Moreover, polysubstance use was common, which may confound the interpretation of SUD-associated virome patterns. In addition, the peripheral comparator pooled PBMC, spleen, and lymph node specimens, which differ biologically and may confound comparisons with brain and CSF. Because brain samples were analyzed previously and matched CSF/peripheral samples were added later, technical or batch-related differences between compartments cannot be excluded. Second, the observational nature of this postmortem analysis restricts conclusions about temporal dynamics, replication competence, or clinical significance of detected viral nucleic acids. Detection alone cannot distinguish between active replication, residual nucleic acid, or transient or compartment-limited detection. Incomplete serologic testing near the time of death further limits classification of HBV detection patterns in some donors. Third, while the ViroFind method enables enhanced detection of low-abundance viral sequences, uneven sequencing depth and read coverage constrained single-nucleotide variant calling in some cases. This may have masked subtle sequence differences across compartments. While salmon sperm DNA was added as carrier nucleic acid for low-yield CSF samples, technical effects on low-biomass CSF detection cannot be fully excluded. Because brain samples were analyzed previously and matched CSF/peripheral samples were added later, technical or batch-related differences between compartments cannot be excluded. Finally, without tissue-level localization or host transcriptomic data, our ability to contextualize viral findings within host immune or neuropathologic processes is limited. These caveats underscore the need for follow-up studies using longitudinal, multi-modal designs with larger cohorts and broader anatomic sampling to confirm and expand upon these initial observations.

Metagenomic sequencing has become an important tool for the clinical diagnosis of CNS infections [1,2,3,4]. However, the marked excess of host nucleic acid over viral nucleic acid may limit sensitivity, making target enrichment an important strategy for improving detection. In a pilot study, ViroFind enriched viral sequences up to 127-fold in human brain samples compared with deep sequencing alone [26]. Our recent IJMS study further showed that blood and CSF viral detection have distinct predictive relationships with brain viral presence, with blood negativity generally being more informative for excluding brain detection and CSF showing limited predictive utility across most taxa [15]. In that context, the present study adds a complementary layer by defining cross-compartment virome discordance within matched brain, CSF, and peripheral samples, and by highlighting HBV as a virus of particular interest for future study. Together, these findings support the value of multi-compartment virome profiling for refining how peripheral and CNS viral signals are interpreted and for guiding future work on the biological and clinical significance of viral detection across compartments.

## 4. Materials and Methods

### 4.1. Postmortem Samples

A total of 24 matching CSF samples of the previous 72 brain samples from the NNTC [14] were available for testing. Among all CSF samples, 8 samples were HIV^+^/SUD^+^, 12 samples were HIV^+^/SUD^−^, 3 samples were HIV^−^/SUD^+^, and only one CSF sample was from HIV^−^/SUD^−^ group. A total of 63 matching spleen samples of the previous 72 brain samples were available for testing. These included all 16/20 samples from the HIV^+^/SUD^+^ group, with sample G1S02 having an additional lymph node (LN) sample, and 19/20 samples from the HIV^+^/SUD^−^ group with an additional LN sample for donor number G2S05. There were 14/16 samples of the HIV^−^/SUD^+^ group, and 14/20 samples of the HIV^−^/SUD^−^ group. In addition, we obtained one unique PBMC sample from donor number G2S08, which allowed us to test 20/20 individuals in the HIV^+^/SUD^−^ group. We also obtained two unique lymph node samples from donor numbers G3S09 and G4S07, allowing us to test 15 individuals of the HIV^−^/SUD^+^ and HIV^−^/SUD^−^ groups. Spleen, LN, and PBMC samples were combined together and named the “Peripheral samples (P)” category for consistency in the figures.

### 4.2. Determination of Substance Use Disorder

SUD diagnoses were determined using the Psychiatric Research Interview for Substance and Mental Disorders (PRISM), a clinician-administered, semi-structured diagnostic interview specifically designed to improve the reliability of psychiatric diagnoses in individuals with substance use [27]. The PRISM assesses both lifetime and current DSM-IV diagnoses of substance abuse and dependence across multiple substance classes, including cocaine, opiates, alcohol, cannabis, sedatives, stimulants, and hallucinogens. The interrater reliability of the PRISM was validated across all the sites of the National NeuroAIDS Tissue Consortium (NNTC) with kappa coefficients for lifetime substance abuse or dependence diagnoses ranging from 0.66 to 1.00, indicating good-to-excellent agreement [28]. PRISM interviews were administered at enrollment and at regular follow-up intervals (every 3–6 months) as part of the NNTC’s comprehensive longitudinal assessment, which also included detailed substance use histories, neuropsychological testing, and neuromedical evaluations [29,30].

### 4.3. IRB Approval

The study was approved by the Institutional Review Board of Northwestern University (STU00211556). Human tissues were obtained from the National NeuroAIDS Tissue Consortium (NNTC). Contributing sites operate under the following IRB-approved protocols: Texas NeuroAIDS Research Center (TNRC), University of Texas Medical Branch, IRB-approved projects 98-402 and 03-195; California NeuroAIDS Tissue Network (CNTN), University of California, San Diego, IRB-approved projects 171024 and 080323; National Neurological AIDS Bank (NNAB), University of California, Los Angeles, IRB-approved project 10-000525; and Manhattan HIV Brain Bank (MHBB), Icahn School of Medicine at Mount Sinai, IRB-approved project HS11-00388. All specimens provided to the investigators were archival and de-identified.

### 4.4. DNA/RNA Extraction from CSF

One ml CSF was used for DNA/RNA extraction with QIAGEN MinElute Virus Spin Kit (QIAGEN, Hilden, Germany; cat. no. 57704). Modified protocol was used as described below: 25 uL QIAGEN protease was added to every 1.7 mL tube, then 200 uL aliquoted CSF was added. Every sample was divided into 5 aliquots (tubes). Then, 200 uL Buffer AL (containing 28 ug/mL carrier RNA) was added to every tube, followed by a 56 °C 15 min incubation, then 250 uL 100% molecular biology grade ethanol was added to every tube. This was followed by standard protocol for the column washing steps with Buffer AW1, AW2, and 100% ethanol, with 2 mL collection tube changes after each washing step. All 5 columns from the same sample were eluted into the same sample using 150 uL buffer AVE in a sequential manner, yielding approximately 110 uL of extracted DNA/RNA.

### 4.5. DNA and RNA Extraction from PBMC

DNA and RNA were extracted from PBMC (2 × 10^5^–4 × 10^6^ total cells) with QIAGEN AllPrep DNA/RNA Universal Kit (QIAGEN, Hilden, Germany; cat. no. 80224) using a modified protocol. PBMC were first washed by 1XPBS, then resuspended with 200 uL 1XPBS, followed by adding 350 uL/600 uL Buffer RLT/ β-2ME/Reagent DX for no more than 3 million PBMC and 3–5 million PBMC, respectively. Then, follow the standard kit protocol. RNA was eluted into 50 uL RNase free water and DNA was eluted with 100 uL Buffer EB.

### 4.6. DNA and RNA Extraction from Spleen and Lymph Node

A total of 20 mg of spleen and 30 mg of lymph node were used for DNA and RNA extraction with QIAGEN AllPrep DNA/RNA Universal Kit (QIAGEN, Hilden, Germany; cat. no. 80224) with standard kit protocol.

### 4.7. ViroFind Design

ViroFind was performed as previously described [14], with some modifications. Due to the low DNA extraction yield of all CSF samples (concentration of the final combined DNA solution was undetectable), 400 ng salmon sperm DNA was spiked into starting material as a carrier nucleic acid to ensure the total starting DNA would reach the 400 ng requirement. To avoid possible index hopping, CSF samples were only processed in the same ViroFind processing batch. Mixing of CSF samples with other high DNA yield samples (spleen, PBMC, or lymph nodes) was carefully avoided.

### 4.8. ViroFind Data Processing

All steps of ViroFind data processing, including sequencing data analysis, curation, thresholding, and statistical analysis, followed the same protocol and standards described previously [14].

### 4.9. Single-Nucleotide Polymorphisms Analysis of Hepatitis B Virus Genome

#### 4.9.1. Criteria of HBV SNPs Selection

Single-nucleotide polymorphism (SNP) analysis was performed to compare Hepatitis B (HBV) sequences across compartments. SNPs were determined only for sequence regions which had coverage from all donor compartments where sequencing depth was ≥5 reads.

#### 4.9.2. Compartmentalization Analysis of HBV Strains

Compartmentalization analysis was performed using allele frequency comparisons derived from variant call format (VCF) files generated for brain, cerebrospinal fluid (CSF), and peripheral compartments. Each sample was processed individually to produce variant-only VCF using bcftools mpileup and bcftools call under haploid assumptions, referencing the HBV-ayw strain (GenBank accession NC_003977.2). Callable regions were defined separately for each sample using coverage masks requiring a minimum depth of five reads per position. All pairwise comparisons were then performed among the three compartments within each donor (brain vs. CSF, brain vs. periphery, and CSF vs. periphery). Comparisons were limited to sites callable in both members of the pair. For each pairwise comparison, per-site summary tables were generated containing the total read depth (DP), reference read count (REF), and alternate read count (ALT) in both compartments. Allele frequency (AF) was calculated as ALT divided by DP for each compartment. Sites were included in downstream analyses only when both compartments met the minimum depth requirement. The allele frequency difference (ΔAF) was calculated as AF_A minus AF_B, and the absolute difference (|ΔAF|) was computed to quantify the magnitude of compartmental divergence. Discordance status was determined for each site according to whether either compartment exhibited an AF greater than or equal to 0.05 while the other compartment was below this threshold. Statistical significance of allele frequency differences was assessed using Fisher’s exact test based on the raw counts of reference and alternate reads in each compartment. All pairwise site-level outputs were merged for each donor into a combined table that preserved block-specific information for brain–CSF, brain–periphery, and CSF–periphery comparisons. Duplicate or ambiguous column names generated during merging were resolved programmatically by appending compartment-specific suffixes prior to concatenation to prevent column collision. Canonical AF and DP values for each compartment were then recomputed directly from ALT and DP counts to eliminate dependency on any previously calculated AF fields. When both brain–CSF and brain–periphery comparisons provided allele frequency estimates for brain, the two values were cross-checked and averaged only when consistent within a tolerance of 1 × 10^−6^. Similar checks were applied for CSF and periphery. For each site, a categorical proximity call was generated based on pairwise allele frequency distances. A site was classified as “brain-closer” if the absolute difference between CSF and brain allele frequencies was smaller than the absolute difference between CSF and periphery by more than 0.01. Conversely, it was classified as “blood-closer” if the CSF–periphery distance was smaller by more than 0.01. If the two distances were within 0.01 of each other, the site was classified as a tie. Sites were excluded from proximity assignment when any compartment had fewer than ten reads of coverage.

Principal component analysis (PCA) was conducted on the absolute allele frequency differences across all callable sites and separately on the subset of informative sites. Informative sites were defined as those with statistically significant Fisher’s exact test results (*p* < 0.05) and an absolute allele frequency difference of at least 0.05. In a secondary analysis, a more stringent informative subset was defined by requiring both conditions to be met simultaneously (*p* < 0.05 and |ΔAF| ≥ 0.10). To visualize overall similarity among compartment-specific HBV variant profiles, a distance-based neighbor-joining tree was generated from the union of variant sites identified across samples. For each sample, alternate allele frequency was calculated from VCF FORMAT fields as alternate allelic depth divided by total allelic depth. A site-by-sample allele frequency matrix was generated, invariant sites were removed, and pairwise sample distances were calculated as the mean Manhattan distance across variable sites. Neighbor-joining was then performed using this allele frequency distance matrix to visualize relative branch lengths among compartment-specific HBV variant profiles. Per-donor summary counts of proximity classifications were computed for both the all-sites matrix and the informative subset, and the ratio of brain-closer to blood-closer sites was used to assess directional bias in CSF similarity. All analyses were performed using a custom Python 3.8 script. The allele frequency profile neighbor-joining tree was generated using a custom Python 3.8 script and plotted in R version 3.6.1. The script utilized pandas for data manipulation, numpy for array operations, matplotlib for figure generation, and scikit-learn for PCA and standard scaling. The minimum depth threshold for inclusion was set to 10 reads for the final analysis, the tie buffer for proximity classification was set to 0.01, and the informative ΔAF thresholds were set at 0.05 and 0.10 for exploratory and confirmatory analyses, respectively.

#### 4.9.3. Statistical Analysis

Statistical analyses were performed in R (version 4.3.1). All analyses and figures were generated using base R functions and the following packages: dplyr (version 1.1.4), and ggplot2 (version 3.4.4). Viral prevalence was compared across compartments or host groups using Fisher’s exact test. Viral burden was compared using non-parametric Wilcoxon rank-sum or Kruskal–Wallis tests when more than two groups were evaluated. Concordance analysis FDR was adjusted for multiple comparisons using the Benjamini–Hochberg procedure. All tests were two-tailed. For compartment-level comparisons of cumulative viral burden (brain, CSF, periphery), we used Kruskal–Wallis tests followed by pairwise Wilcoxon tests. To assess CNS–periphery viral discordance, we calculated the probability of CNS detection, conditional on peripheral detection, and estimated risk differences, risk ratios, and exact 95% confidence intervals using Fisher’s exact test-derived contingency tables. Host factor analyses (HIV^+^ vs. HIV^−^ and SUD^+^ vs. SUD^−^) were performed independently within each compartment using the same prevalence and burden protocols described above. Benjamini–Hochberg correction was applied to the concordance analyses in Table 3; all other taxon-level group comparisons and HBV sequence-comparison analyses were treated as exploratory [31].

## Figures and Tables

**Table 1:** Summary of substance use characteristics among SUD^+^ participants.

	Urine Screening				Psychiatric Diagnosis (PRISM)			
	Overall (*n* = 27)	HIV^+^/SUD^+^ (*n* = 14)	HIV^−^/SUD^+^ (*n* = 13)	*p*	Overall (*n* = 23)	HIV^+^/SUD^+^ (*n* = 16)	HIV^−^/SUD^+^ (*n* = 7)	*p*
No. of substances/subject	1 [1–3]	2 [1–3]	1 [1–1]	**0.002**	3 [2–3]	3 [2–3.5]	2 [1–3]	0.192
(median [IQR])								
Substance % (*n*)								
Cannabis	37.0 (10)	57.1 (8)	2	**0.046**	47.8 (11)	62.5 (10)	14.3 (1)	0.069
Opiates								
Unspecified	37.0 (10)	42.9 (6)	4	0.695	39.1 (9)	50.0 (8)	14.3 (1)	0.176
Methadone	18.5 (5)	0 (0)	5	**0.041**	N/A	N/A	N/A	
Stimulants								
Unspecified	N/A	N/A	N/A		17.4 (4)	18.8 (3)	14.3 (1)	1
Methamphetamine	7.4 (2)	14.3 (2)	0 (0)	0.482	N/A	N/A	N/A	
Cocaine	44.4 (12)	78.6 (11)	1	**<0.001**	65.2 (15)	75 (12)	42.9 (3)	0.182
Sedatives								
Unspecified	N/A	N/A	N/A		21.7 (5)	31.3 (5)	0 (0)	0.272
Benzodiazepine	25.9 (7)	28.6 (4)	3	1	N/A	N/A	N/A	
Barbiturates	3.7 (1)	7.1 (1)	0 (0)	1	N/A	N/A	N/A	
Hallucinogens								
Unspecified	N/A	N/A	N/A		4.3 (1)	6.3 (1)	0 (0)	1
PCP	7.4 (2)	14.3 (2)	0 (0)	0.482	N/A	N/A	N/A	
EtOH	N/A	N/A	N/A		52.2 (12)	56.3 (9)	42.9 (3)	0.667
Other	7.4 (2)	14.3 (2)	0 (0)	0.482	4.3 (1)	0 (0)	14.3 (1)	0.304

No.: number; IQR: interquartile range; PCP: phencyclidine; EtOH: ethanol; PRISM: Psychiatric Research Interview for Substance and Mental Disorders. Significance indicated by emboldened *p*-value (*p* < 0.05).

**Table 2:** Viral prevalence and burden in brain, CSF, and periphery.

	Viral Prevalence				Viral Burden			
	Brain	CSF	Periphery	*p*-Value	Brain	CSF	Periphery	*p*-Value
	Median viral species per subject (IQR)				Median viral rPM per subject (IQR)			
	4.5 (3.3–6)	2.5 (1–3)	6 (5–6.75)	**<0.001**	112.7 (40.4–1234.3)	3.8 (0.8–11.8)	459.4 (63.1–4633.8)	**<0.001**
Viral taxa	*n* (%)				Median rPM			
Human adenovirus	16 (66.7%)	0	0	**<0.001**	0.8 (0.5–1.9)	N/A	N/A	N/A
Torque teno virus	17 (70.8%)	14 (58.3%)	24 (100%)	**<0.001**	9.9 (2.0–56.7)	1.7 (0.6–3.5)	73.6 (6.4–2104.3)	**<0.001**
Hepatitis C virus	0	1 (4.2%)	3 (12.5%)	0.314	N/A	0.4 (N/A)	1.7 (0.2–2.1)	N/A
Human pegivirus	1 (4.2%)	0	3 (12.5%)	0.314	1.3 (N/A)	N/A	0.4 (0.2–12.5)	N/A
Sewage-associated gemycircularvirus	0	1 (4.2%)	0	1	N/A	0.1 (N/A)	N/A	N/A
Hepatitis B virus	3 (12.5%)	8 (33.3%)	2 (8.3%)	0.099	2.9 (0.4–156.9)	0.2 (0.1–7.6)	378.8 (N/A)	N/A
Herpes simplex virus (HHV1)	1 (4.2%)	0	2 (8.3%)	0.768	1.0 (N/A)	N/A	2.3 (N/A)	N/A
Herpes simplex virus (HHV2)	1 (4.2%)	0	0	1	0.5 (N/A)	N/A	N/A	N/A
Varicella zoster virus (HHV3)	0	0	2 (8.3%)	0.324	N/A	N/A	0.3 (N/A)	N/A
Epstein–Barr virus (HHV4)	10 (41.7%)	6 (25.0%)	23 (95.8%)	**<0.001**	6.5 (0.6–53.9)	3.6 (1.4–11.2)	11.6 (3.4–45.6)	0.357
Cytomegalovirus (HHV5)	17 (70.8%)	5 (20.8%)	17 (70.8%)	**<0.001**	5.7 (1.4–19.7)	0.2 (0.2–6.8)	7.36 (0.9–48.3)	0.177
Human herpesvirus 6 A/B	4 (16.7%)	1 (4.2%)	13 (54.2%)	**<0.001**	0.4 (0.2–1.8)	0.2 (N/A)	0.5 (0.3–1.7)	N/A
Human herpesvirus 7	1 (4.2%)	2 (8.3%)	12 (50.0%)	**<0.001**	3.0 (N/A)	0.2 (N/A)	0.4 (0.2–0.8)	N/A
Human herpesvirus 8	0	0	3 (12.5%)	0.102	N/A	N/A	0.87 (0.1–35.6)	N/A
Sphinx1.76-related DNA	10 (41.7%)	3 (12.5%)	4 (16.7%)	**0.039**	1.8 (0.4–4.2)	0.2 (0.2–127.7)	0.3 (0.1–0.6)	0.122
Human papillomavirus	4 (16.7%)	7 (29.2%)	2 (8.3%)	0.204	1.5 (0.3–3.9)	0.7 (0.6–1.4)	0.4 (N/A)	N/A
Adeno-associated virus	3 (12.5%)	0	1 (4.2%)	0.314	1.0 (0.4–2.7)	N/A	0.3 (N/A)	N/A
Human parvovirus	7 (29.2%)	2 (8.3%)	14 (58.3%)	**0.001**	0.5 (0.3–2.6)	0.4 (N/A)	0.6 (0.3–2.7)	N/A
Respiratory syncytial virus (RSV)	0	0	1 (4.2%)	1	N/A	N/A	0.3 (N/A)	N/A
BK polyomavirus (HpyV1)	2 (8.3%)	0	1 (4.2%)	0.768	54.5 (N/A)	N/A	0.2 (N/A)	N/A
Human polyomavirus 6 (HPyV6)	0	0	1 (4.2%)	1	N/A	N/A	0.6 (N/A)	N/A
JC polyomavirus (HPyV2)	9 (37.5%)	2 (8.3%)	0	**<0.001**	1.4 (0.7–444.2)	0.6 (N/A)	N/A	N/A
Simian virus 40	0	1 (4.2%)	0	1	N/A	0.6 (N/A)	N/A	N/A
STL polyomavirus (HPyV11)	0	1 (4.2%)	0	1	N/A	0.2 (N/A)	N/A	N/A
WU polyomavirus (HPyV4)	1 (4.2%)	0	1 (4.2%)	1	10.9 (N/A)	N/A	68.3 (N/A)	N/A
Merkel cell polyomavirus (HPyV5)	0	2 (8.3%)	1 (4.2%)	0.768	N/A	1.1 (N/A)	0.2 (N/A)	N/A
Human immunodeficiency virus	9 (37.5%)	8 (33.3%)	15 (62.5%)	0.100	2.0 (0.4–26.5)	0.8 (0.4–7.0)	4.2 (1.1–31.4)	0.130

IQR: interquartile range; rPM: reads per million; NA: not applicable. Significance indicated by emboldened *p*-value (*p* < 0.05).

**Table 3:** Concordance and discordance of virus detection in CNS and periphery compartments.

Virus	Concordance: Probability of CNS+ if Periphery+	Discordance: Probability of CNS+ if Periphery−	Risk Difference	Risk Ratio	Padj Value
Adeno-associated virus—spp.	1/2 (50%)	4/64 (6%)	44%	8.00	0.269
Hepatitis B virus	3/3 (100%)	8/63 (13%)	87%	7.88	**0.013**
Hepatitis C virus	5/11 (45%)	0/55 (0%)	45%	Infinite	**<0.001**
HHV4-Epstein–Barr virus	18/53 (34%)	2/13 (15%)	19%	2.21	0.432
HHV5-Cytomegalovirus	32/38 (84%)	16/28 (57%)	27%	1.47	0.067
HHV6-human herpesvirus 6A/B	6/38 (16%)	3/28 (11%)	5%	1.47	0.722
Human immunodeficiency virus	14/26 (54%)	4/40 (10%)	44%	5.38	**<0.001**
Human parvovirus spp.	11/34 (32%)	6/32 19%	14%	1.73	0.416
Human pegivirus	1/3 (33%)	11/63 (17%)	16%	1.91	0.504
Sphinx1.76-related DNA	6/9 (67%)	16/57 (28%)	39%	2.38	0.111
Torque teno virus spp.	37/61 (61%)	2/5 (40%)	1%	1.52	0.480

Significance indicated by emboldened *p*-value (*p* < 0.05).

**Table 4:** Summary of all HBV DNA + compartments in NNTC cohort.

Research ID	Brain	CSF	Periphery
G1S01	POS	POS	POS
G1S02	POS	POS	POS
G1S03	NEG	POS	NEG
G1S04	POS	POS	NEG
G1S12	POS	N/A	POS
G2S01	POS	N/A	NEG
G2S02	NEG	POS	NEG
G2S03	NEG	POS	NEG
G2S10	NEG	POS	NEG
G3S04	NEG	POS	NEG
G4S12	POS	N/A	NEG
Sample count	6	8	3
Patient count	11		3

NEG: negative; POS: positive; N/A: not available.

**Table 5:** Viral prevalence and burden in peripheral samples of HIV^+^ vs. HIV^−^.

	Viral Presence			Viral Burden		
	31 SUD^+^	35 SUD^−^	*p*-Value	31 SUD^+^	35 SUD^−^	*p*-Value
	Median viral species per subject (IQR)			Median viral rPM per subject (IQR)		
	5 (4–7)	5 (4–6)	0.578	66.9 (14.52–562.5)	79.1 (8.4–2097.6)	0.878
Viral taxa	*n* (%)			Median rPM		
Torque teno virus	30 (96.8%)	34 (97.1%)	1	11.2 (0.5–172.8)	22.4 (1.1–430.4)	0.435
Human coronavirus NL63	0	1 (2.9%)	1	N/A	0.2 (N/A)	N/A
Human coronavirus OC43	1 (3.2%)	0	0.470	0.1 (N/A)	N/A	N/A
Hepatitis C Virus	10 (32.3%)	1 (2.9%)	**0.002**	1.8 (0.3–36.1)	0.8 (N/A)	N/A
Human pegivirus	1 (3.2%)	2 (5.7%)	1	0.4 (N/A)	6.3 (N/A)	N/A
Hepatitis B Virus	3 (9.7%)	0	0.098	747.1 (10.4–922.1)	N/A	N/A
Herpes simplex virus 1 (HHV1)	4 (12.9%)	1 (2.9%)	0.179	1.5 (0.9–2.6)	1.7 (N/A)	N/A
Herpes simplex virus 2 (HHV2)	1 (3.2%)	1 (2.9%)	1	0.1 (N/A)	0.9 (N/A)	N/A
Varicella zoster virus (HHV3)	0	2 (5.7%)	0.494	N/A	0.3 (N/A)	N/A
Epstein–Barr virus (HHV4)	23 (74.2%)	33 (94.3%)	**0.037**	13.8 (3.1–101.3)	7.4 (1.7–56.6)	0.830
Cytomegalovirus (HHV5)	18 (58.1%)	21 (60%)	1	0.5 (0.2–8.1)	5.2 (0.6–48.3)	0.052
Human herpesvirus 6 A/B	17 (54.8%)	21 (60%)	0.804	0.7 (0.2–1.1)	1.3 (0.4–2.6)	0.159
Human herpesvirus 7	24 (77.4%)	18 (51.4%)	**0.041**	0.7 (0.4–1.8)	0.6 (0.3–1.2)	0.438
Human herpesvirus 8	0	4 (11.4%)	0.116	N/A	0.9 (0.3–27.0)	N/A
Norovirus	0	1 (2.9%)	1	N/A	0.1 (N/A)	N/A
Sphinx 1.76-related DNA	5 (16.1%)	4 (11.4%)	0.724	0.1 (0.1–0.5)	0.2 (0.1–0.3)	1
Human papillomavirus	2 (6.5%)	2 (5.7%)	1.000	0.3 (N/A)	0.2 (N/A)	N/A
Human parainfluenza virus type 3	1 (3.2%)	1 (2.9%)	1	0.26 (N/A)	11 (N/A)	N/A
Adeno-associated virus	2 (6.5%)	0	0.217	0.3 (N/A)	N/A	N/A
Human parvovirus	17 (54.8%)	17 (48.6%)	0.631	0.3 (N/A)	1.1 (0.4–2.3)	N/A
Respiratory syncytial virus (RSV)	0	1 (2.9%)	1	0.4 (0.2–4.6)	0.3 (N/A)	N/A
BK polyomavirus (HPyV1)	0	2 (5.7%)	0.494	N/A	0.2 (N/A)	N/A
Human polyomavirus 6 (HPyV6)	0	2 (5.7%)	0.494	N/A	0.4 (N/A)	N/A
Merkel cell polyomavirus (HPyV5)	1 (3.2%)	2 (5.7%)	1	0.2 (N/A)	0.2 (N/A)	N/A
WU polyomavirus (HPyV4)	0	1 (2.9%)	1	N/A	68.3 (N/A)	N/A
Human immunodeficiency virus	14 (45.1%)	13 (37.1%)	0.618	1.8 (0.2–65.4)	N/A	N/A
Human T-lymphotropic virus 2	1 (3.2%)	0	1	3.0 (N/A)	4.5 (0.5–55.7)	N/A

IQR: interquartile range; rPM: reads per million; N/A: not applicable. Significance indicated by emboldened *p*-value (*p* < 0.05).

**Table 6:** Viral prevalence and burden in peripheral samples of SUD^+^ vs. SUD^−^.

	Viral Presence			Viral Burden		
	31 SUD^+^	35 SUD^−^	*p*-Value	31 SUD^+^	35 SUD^−^	*p*-Value
	Median viral species per subject (IQR)			Median viral rPM per subject (IQR)		
	5 (4–7)	5 (4–6)	0.578	66.9 (14.52–562.5)	79.1 (8.4–2097.6)	0.878
Viral taxa	*n* (%)			Median rPM		
Torque teno virus	30 (96.8%)	34 (97.1%)	1	11.2 (0.5–172.8)	22.4 (1.1–430.4)	0.435
Human coronavirus NL63	0	1 (2.9%)	1	N/A	0.2 (N/A)	N/A
Human coronavirus OC43	1 (3.2%)	0	0.470	0.1 (N/A)	N/A	N/A
Hepatitis C Virus	10 (32.3%)	1 (2.9%)	**0.002**	1.8 (0.3–36.1)	0.8 (N/A)	N/A
Human pegivirus	1 (3.2%)	2 (5.7%)	1	0.4 (N/A)	6.3 (N/A)	N/A
Hepatitis B Virus	3 (9.7%)	0	0.098	747.1 (10.4–922.1)	N/A	N/A
Herpes simplex virus 1 (HHV1)	4 (12.9%)	1 (2.9%)	0.179	1.5 (0.9–2.6)	1.7 (N/A)	N/A
Herpes simplex virus 2 (HHV2)	1 (3.2%)	1 (2.9%)	1	0.1 (N/A)	0.9 (N/A)	N/A
Varicella zoster virus (HHV3)	0	2 (5.7%)	0.494	N/A	0.3 (N/A)	N/A
Epstein–Barr virus (HHV4)	23 (74.2%)	33 (94.3%)	**0.037**	13.8 (3.1–101.3)	7.4 (1.7–56.6)	0.830
Cytomegalovirus (HHV5)	18 (58.1%)	21 (60%)	1	0.5 (0.2–8.1)	5.2 (0.6–48.3)	0.052
Human herpesvirus 6 A/B	17 (54.8%)	21 (60%)	0.804	0.7 (0.2–1.1)	1.3 (0.4–2.6)	0.159
Human herpesvirus 7	24 (77.4%)	18 (51.4%)	**0.041**	0.7 (0.4–1.8)	0.6 (0.3–1.2)	0.438
Human herpesvirus 8	0	4 (11.4%)	0.116	N/A	0.9 (0.3–27.0)	N/A
Norovirus	0	1 (2.9%)	1	N/A	0.1 (N/A)	N/A
Sphinx 1.76-related DNA	5 (16.1%)	4 (11.4%)	0.724	0.1 (0.1–0.5)	0.2 (0.1–0.3)	1
Human papillomavirus	2 (6.5%)	2 (5.7%)	1.000	0.3 (N/A)	0.2 (N/A)	N/A
Human parainfluenza virus type 3	1 (3.2%)	1 (2.9%)	1	0.26 (N/A)	11 (N/A)	N/A
Adeno-associated virus	2 (6.5%)	0	0.217	0.3 (N/A)	N/A	N/A
Human parvovirus	17 (54.8%)	17 (48.6%)	0.631	0.3 (N/A)	1.1 (0.4–2.3)	N/A
Respiratory syncytial virus (RSV)	0	1 (2.9%)	1	0.4 (0.2–4.6)	0.3 (N/A)	N/A
BK polyomavirus (HPyV1)	0	2 (5.7%)	0.494	N/A	0.2 (N/A)	N/A
Human polyomavirus 6 (HPyV6)	0	2 (5.7%)	0.494	N/A	0.4 (N/A)	N/A
Merkel cell polyomavirus (HPyV5)	1 (3.2%)	2 (5.7%)	1	0.2 (N/A)	0.2 (N/A)	N/A
WU polyomavirus (HPyV4)	0	1 (2.9%)	1	N/A	68.3 (N/A)	N/A
Human immunodeficiency virus	14 (45.1%)	13 (37.1%)	0.618	1.8 (0.2–65.4)	N/A	N/A
Human T-lymphotropic virus 2	1 (3.2%)	0	1	3.0 (N/A)	4.5 (0.5–55.7)	N/A

IQR: interquartile range; rPM: reads per million; N/A: not applicable. Significance indicated by emboldened *p*-value (*p* < 0.05).

**Table 7:** Viral prevalence and burden in CSF samples of SUD^+^ vs. SUD^−^.

	Viral Presence				Viral Burden			
	Brain	CSF	Periphery	*p*-Value	Brain	CSF	Periphery	*p*-Value
	Median viral species per subject (IQR)				Median viral rPM per subject (IQR)			
	4.5 (3.3–6)	2.5 (1–3)	6 (5–6.75)	**<0.001**	112.7 (40.4–1234.3)	3.8 (0.8–11.8)	459.4 (63.1–4633.8)	**<0.001**
Viral taxa	*n* (%)				Median rPM			
Human adenovirus	16 (66.7%)	0	0	**<0.001**	0.8 (0.5–1.9)	N/A	N/A	N/A
Torque teno virus	17 (70.8%)	14 (58.3%)	24 (100%)	**<0.001**	9.9 (2.0–56.7)	1.7 (0.6–3.5)	73.6 (6.4–2104.3)	**<0.001**
Hepatitis C virus	0	1 (4.2%)	3 (12.5%)	0.314	N/A	0.4 (N/A)	1.7 (0.2–2.1)	N/A
Human pegivirus	1 (4.2%)	0	3 (12.5%)	0.314	1.3 (N/A)	N/A	0.4 (0.2–12.5)	N/A
Sewage-associated gemycircularvirus	0	1 (4.2%)	0	1	N/A	0.1 (N/A)	N/A	N/A
Hepatitis B virus	3 (12.5%)	8 (33.3%)	2 (8.3%)	0.099	2.9 (0.4–156.9)	0.2 (0.1–7.6)	378.8 (N/A)	N/A
Herpes simplex virus (HHV1)	1 (4.2%)	0	2 (8.3%)	0.768	1.0 (N/A)	N/A	2.3 (N/A)	N/A
Herpes simplex virus (HHV2)	1 (4.2%)	0	0	1	0.5 (N/A)	N/A	N/A	N/A
Varicella zoster virus (HHV3)	0	0	2 (8.3%)	0.324	N/A	N/A	0.3 (N/A)	N/A
Epstein–Barr virus (HHV4)	10 (41.7%)	6 (25.0%)	23 (95.8%)	**<0.001**	6.5 (0.6–53.9)	3.6 (1.4–11.2)	11.6 (3.4–45.6)	0.357
Cytomegalovirus (HHV5)	17 (70.8%)	5 (20.8%)	17 (70.8%)	**<0.001**	5.7 (1.4–19.7)	0.2 (0.2–6.8)	7.36 (0.9–48.3)	0.177
Human herpesvirus 6 A/B	4 (16.7%)	1 (4.2%)	13 (54.2%)	**<0.001**	0.4 (0.2–1.8)	0.2 (N/A)	0.5 (0.3–1.7)	N/A
Human herpesvirus 7	1 (4.2%)	2 (8.3%)	12 (50.0%)	**<0.001**	3.0 (N/A)	0.2 (N/A)	0.4 (0.2–0.8)	N/A
Human herpesvirus 8	0	0	3 (12.5%)	0.102	N/A	N/A	0.87 (0.1–35.6)	N/A
Sphinx1.76-related DNA	10 (41.7%)	3 (12.5%)	4 (16.7%)	**0.039**	1.8 (0.4–4.2)	0.2 (0.2–127.7)	0.3 (0.1–0.6)	0.122
Human papillomavirus	4 (16.7%)	7 (29.2%)	2 (8.3%)	0.204	1.5 (0.3–3.9)	0.7 (0.6–1.4)	0.4 (N/A)	N/A
Adeno-associated virus	3 (12.5%)	0	1 (4.2%)	0.314	1.0 (0.4–2.7)	N/A	0.3 (N/A)	N/A
Human parvovirus	7 (29.2%)	2 (8.3%)	14 (58.3%)	**0.001**	0.5 (0.3–2.6)	0.4 (N/A)	0.6 (0.3–2.7)	N/A
Respiratory syncytial virus (RSV)	0	0	1 (4.2%)	1	N/A	N/A	0.3 (N/A)	N/A
BK polyomavirus (HpyV1)	2 (8.3%)	0	1 (4.2%)	0.768	54.5 (N/A)	N/A	0.2 (N/A)	N/A
Human polyomavirus 6 (HPyV6)	0	0	1 (4.2%)	1	N/A	N/A	0.6 (N/A)	N/A
JC polyomavirus (HPyV2)	9 (37.5%)	2 (8.3%)	0	**<0.001**	1.4 (0.7–444.2)	0.6 (N/A)	N/A	N/A
Simian virus 40	0	1 (4.2%)	0	1	N/A	0.6 (N/A)	N/A	N/A
STL polyomavirus (HPyV11)	0	1 (4.2%)	0	1	N/A	0.2 (N/A)	N/A	N/A
WU polyomavirus (HPyV4)	1 (4.2%)	0	1 (4.2%)	1	10.9 (N/A)	N/A	68.3 (N/A)	N/A
Merkel cell polyomavirus (HPyV5)	0	2 (8.3%)	1 (4.2%)	0.768	N/A	1.1 (N/A)	0.2 (N/A)	N/A
Human immunodeficiency virus	9 (37.5%)	8 (33.3%)	15 (62.5%)	0.100	2.0 (0.4–26.5)	0.8 (0.4–7.0)	4.2 (1.1–31.4)	0.130

IQR: interquartile range; rPM: reads per million; N/A: not applicable. Significance indicated by emboldened *p*-value (*p* < 0.05).

## Data Availability

The original contributions presented in this study are included in the article/Supplementary Materials. Further inquiries can be directed to the corresponding author.

## Supplementary Materials

The following supporting information can be downloaded at: https://www.mdpi.com/article/10.3390/ijms27125349/s1.
