# Supplementary material for: Cross-Compartment Virome Profiling in Human Immunodeficiency Virus Infection and Substance Use Disorder Reveals Brain–CSF–Periphery Discordance and Hepatitis B Virus in Central Nervous System
Source: Int J Mol Sci. 2026 Jun 13;27(12):5349. doi: 10.3390/ijms27125349 (PMC13300052; doi:10.3390/ijms27125349)

Figure S1: Virome findings by compartment

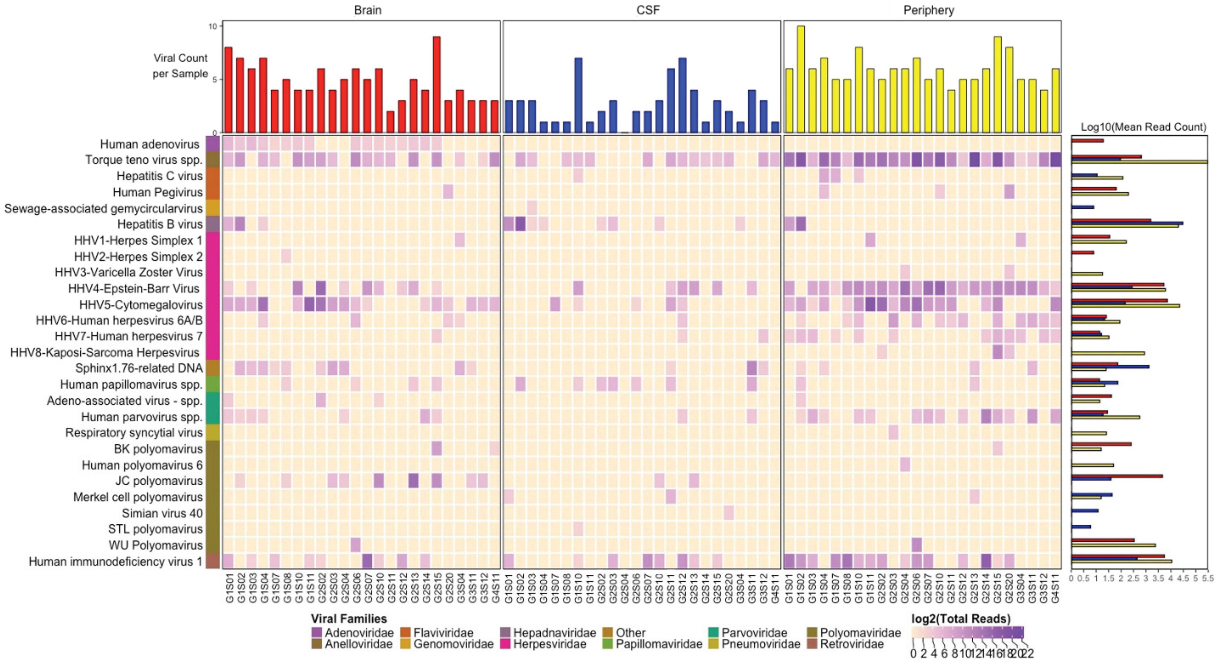

Figure S2: Peripheral virome by HIV status

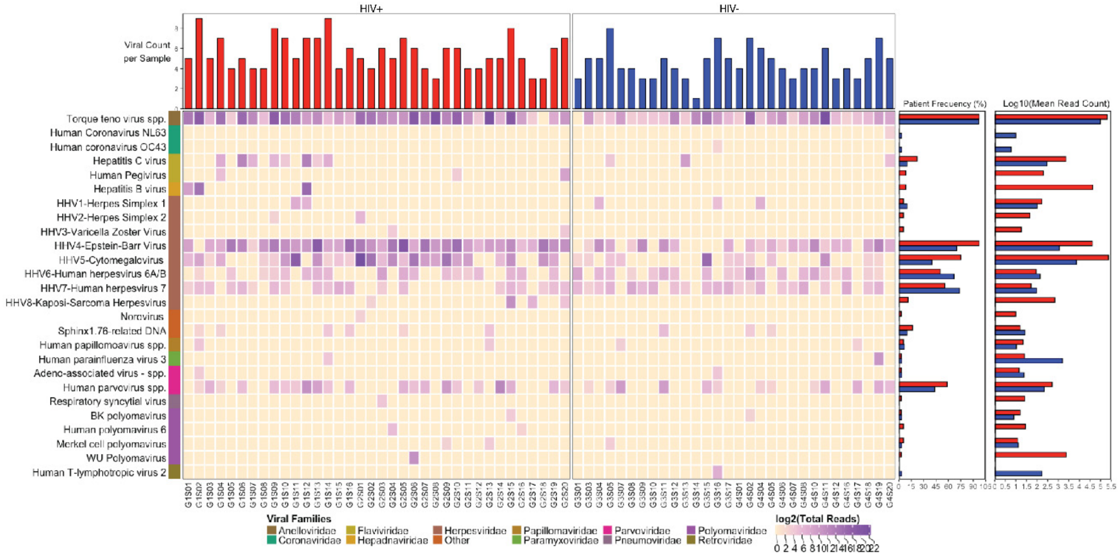

Figure S3: Peripheral virome by SUD status

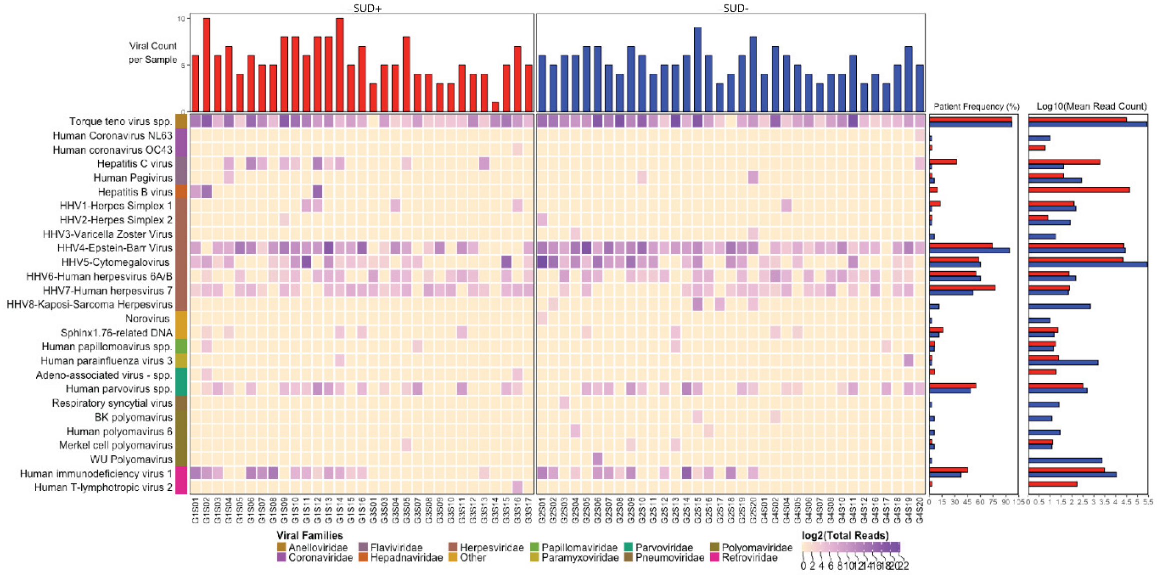

Figure S4: CSF virome by SUD status

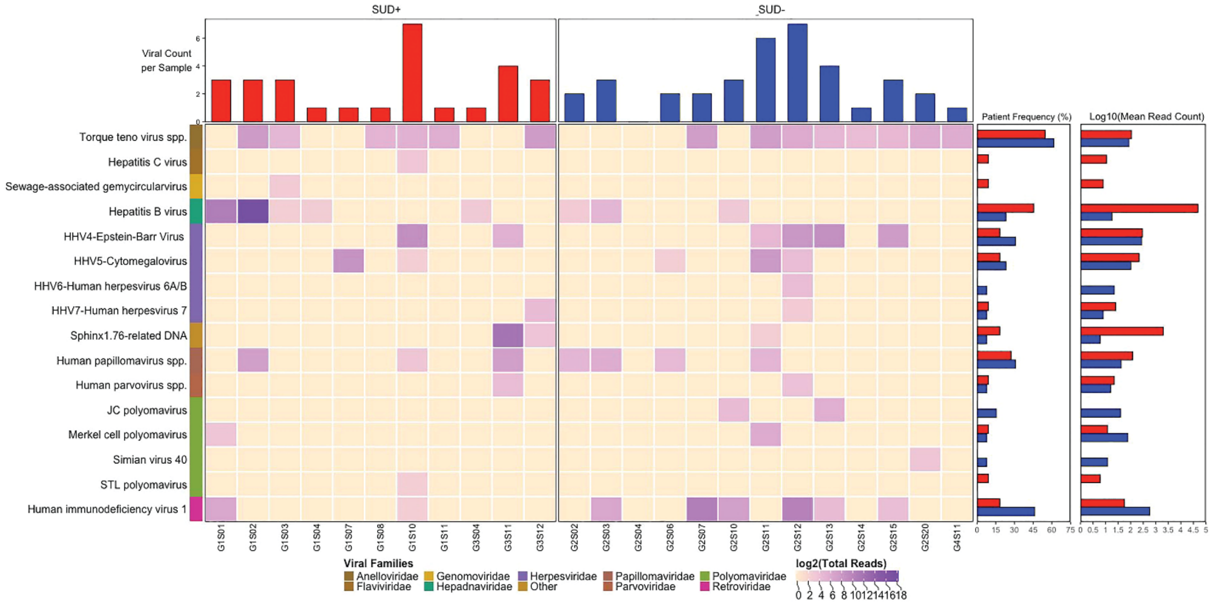

Supplement: Supplementary file 1 [file ijms-27-05349-s001.zip › Supplemental Figures.pdf]
