# Supplementary figures and images for: Cross-Compartment Virome Profiling in Human Immunodeficiency Virus Infection and Substance Use Disorder Reveals Brain–CSF–Periphery Discordance and Hepatitis B Virus in Central Nervous System
Source: Int J Mol Sci. 2026 Jun 13;27(12):5349. doi: 10.3390/ijms27125349 (PMC13300052; doi:10.3390/ijms27125349)

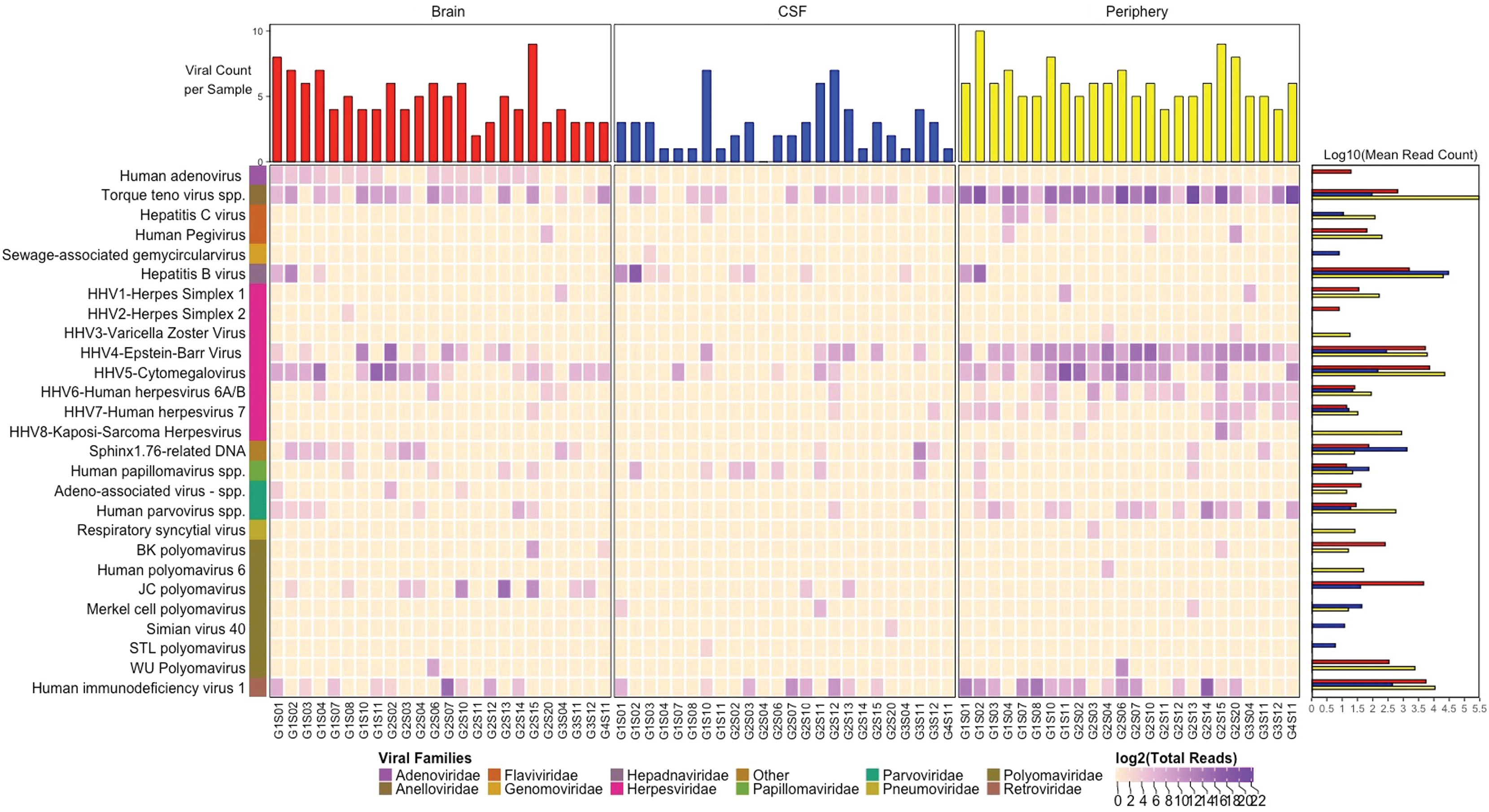

Supplement: Supplementary file 1 [file ijms-27-05349-s001.zip › Figure S1.tif]
